# Liquid Nitrous Oxide

**Published:** 1872-08

**Authors:** 


*Liquid Nitrous Oxide.*—Dr. Morrison in the "Missouri Dental Journal," May number, gives his experience with this preparation as follows:

"We have been using liquid nitrous oxide gas almost daily for the past few months, and do now unhesitatingly pronounce it the best anæsthetic we have ever used. We have tried it for extracting teeth, for minor surgical operations, and especially for excavating sensitive dentine. We will give just one case. A lady, about twenty-two years of age, nervo-bilious temperament, had a natural repugnance and cultivated hatred for dental treatment. Her husband was very anxious to have her teeth put in thorough repair. There were six large, but not deep, cavities of decay in the superior incisors, and a dozen more in the bicuspids and molars. On the most gentle touch with an excavator to the decayed portion of a tooth, she would make a spasmodic jerk, or thrust at the hand of the operator, which would send the instrument hurling in the air. By giving the patient three or four inhalations of gas, we were enabled to entirely remove all the sensitive dentine by a few vigorous cuts of the excavator, and the whole painful part of the operation was completed, leaving only the smoothing of the borders of the cavity and the filling, and finishing the gold, the part of the operation of which she had no dread. We have had a good deal of experience with *ordinary* gas, and find that one half the quantity of *liquid* gas will produce the same anæsthetic effect, and the latter is always fresh and ready for use in a neat and convenient form."

The apparatus of Johnston Bros., consists of an iron cylinder, twelve and a half inches long by three inches in diameter, into which one hundred gallons of the gas are forced by compression. A leather case contains the cylinder and protects the delicate valve with which it is provided. When preparing the apparatus for use, the gas is drawn off into an india-rubber bag capable of holding from four to six gallons and having a suitable inhaler.

